# Impact of Japan’s State of Emergency Due to the COVID-19 Pandemic on Trends in Diabetes Care: A Descriptive and Retrospective Study

**DOI:** 10.3390/clinpract13010013

**Published:** 2023-01-16

**Authors:** Akira Minoura, Kouzou Murakami, Masaaki Matoba, Yoshinori Ito, Yumi Kamijo, Akatsuki Kokaze

**Affiliations:** 1 Department of Hygiene, Public Health and Preventive Medicine, School of Medicine, Showa University, Tokyo 142-8555, Japan; 2 Department of Radiology, Division of Radiology, School of Medicine, Showa University, Tokyo 142-8555, Japan; 3 Department of Health Management, Graduate School of Health Sciences, Showa University, Tokyo 142-8555, Japan

**Keywords:** COVID-19 pandemic, diabetes care, infection control, Japanese, state of emergency

## Abstract

Objective: This study examined the impact of Japan’s state of emergency on trends in diabetes care during the coronavirus disease 2019 (COVID-19) pandemic. Design: A descriptive and retrospective study. Setting: Showa University Hospital, Japan. Participants: Patients with diabetes who received medical treatment from 2018 to 2020. Determinants of interest: Number of patients with diabetes visiting the hospital per week. To examine the impact of the Japan’s state of emergency, the number of hospital visitations by patients with diabetes was summarized from 28 weeks of data for each year, from calendar week 8 to calender week 35. Results: Compared with the mean of 2018 and 2019, no significant difference was found between the three periods (before, during, and after the state of emergency). However, the numbers of patients from both inside and outside Tokyo increased at 7 weeks after the state of emergency was lifted. Conclusions: A significant increase in the numbers of patients with diabetes was seen compared with the same period in 2018 and 2019, suggesting that the state of emergency may have hindered diabetes care. Therefore, patients with diabetes should receive continuous follow-up regarding their diabetes care, keeping a close eye on relvent measurements.

## 1. Introduction

Worldwide, the coronavirus 2019 (COVID-19) pandemic has caused a continuous global public health emergency since 2020 [1]. In this pandemic, substantial increases in infected and critically ill patients resulted in shortages of medical staff and resources, intensive care units, ventilators, and personal protective equipment. Many countries implemented lockdowns, including service closures and restrictions on outings, to stem the spread of COVID-19. The lockdowns in most countries compelled compliance by force and penalized violations. The lockdowns prevent the spread of infection, which would would otherwise have been devastating, leading to economic damage as well as delays in treatment [2]. Delayed diabetes care was a particular problem in health systems worldwide. Ikesu et al. reported that the number of diabetes care services in Japan declined in the weeks following the outbreak of COVID-19 [3].

During the state of emergency, Japan chose to enforce a “mild lockdown,” which was unenforceable and nonpunitive [4]. On 7 April 2020, the government declared that Japan was in a state of emergency due to the outbreak in seven prefectures (urban areas). Tokyo had the highest number of infected patients among all prefectures, and the Tokyo Metropolitan Government asked its citizens to impose their own restrictions. [5]. The state of emergency expanded throughout Japan on 16 April 2020, and was gradually lifted after 14 May 2020. The COVID-19 pandemic policy in Japan was characterized as “requesting” citizens to refrain from going out, except for emergencies, and to temporarily close certain businesses. The pandemic transformed citizens’ activities and medical treatment throughout Japan. For example, the number of monthly train users decreased by 45.5% in April 2020 compared with in April 2019 [6]. Japanese patients with chronic diseases were more likely to become severely ill from COVID-19 infection, and it is possible that patients with diabetes may have been discouraged from seeing a doctor [7]. Therefore, examining the COVID-19 pandemic’s impact on diabetes care is a critical issue, as this affected the mortality rate in Japan.

We have already demonstrated how the state of emergency may have affected medical examinations for the three major diseases in Japan [8]. Yagome et al. showed that the COVID-19 pandemic suppressed physician visits and led to an increase in telemedicine use among diabetic patients in Japan from April to May 2020 [9]. However, there could be a difference in impact between areas with high and low numbers of COVID-19-infected patients. Terakawa et al. showed that work, including telework, affected glycemic control among diabetic patients in Japan [10]. In this regard, it is important to analyze the environmental characteristics of diabetic patients during the pandemic.

Against this background, the present study, which hypothesizes that Japan’s state of emergency suppressed patient hospital visits for diabetes care, aimed to compare trends in consultations for diabetes patients in Tokyo during the state of emergency, and to examine changes in patient consultations, including transfers across prefectures.

## 2. Methods

**Figure 1:**
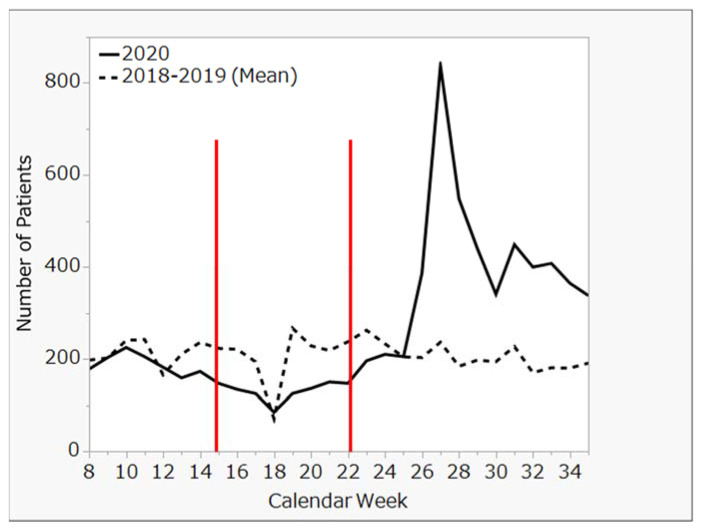
Trends in number of diabetes patients from Tokyo from 2018 through 2020.

**Figure 2:**
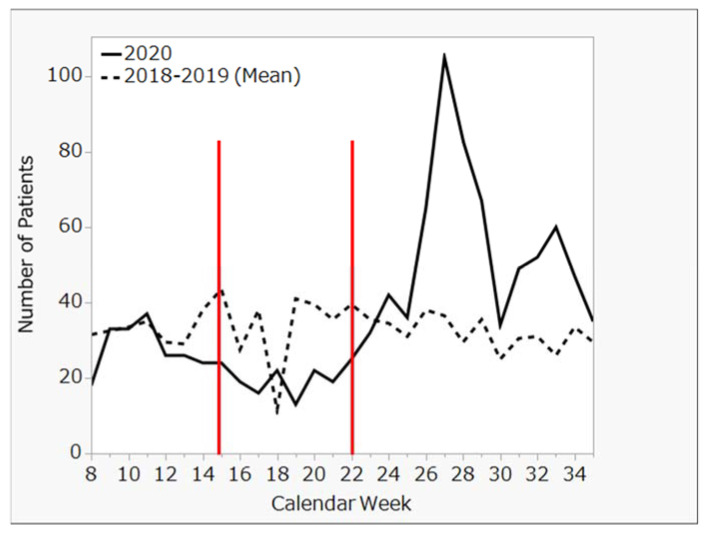
Trends in number of diabetes patients from outside Tokyo from 2018 through 2020.

This retrospective and descriptive study was carried out in Tokyo. The study participants were patients with diabetes, who were diagnosed at Showa University Hospital and received medical treatment from 2018 to 2020. To examine the impact of Japan’s state of emergency, the number of visits to Showa University Hospital for diabetes were summarized from 28 weeks of data, from calendar week 8 to calendar week 35 (as shown in Figure 1 and Figure 2). Showa University Hospital is located in eastern Tokyo, near the border of Kanagawa, with a secondary populous prefecture. There was a high number of COVID-19 patients in this area from early stages of the pandemic, indicating that this is one of areas that were most affected by the COVID-19 pandemic. If participants were returning to the hospital, they were defined as patients with a newly diagnosed disease.

**Figure 3:**
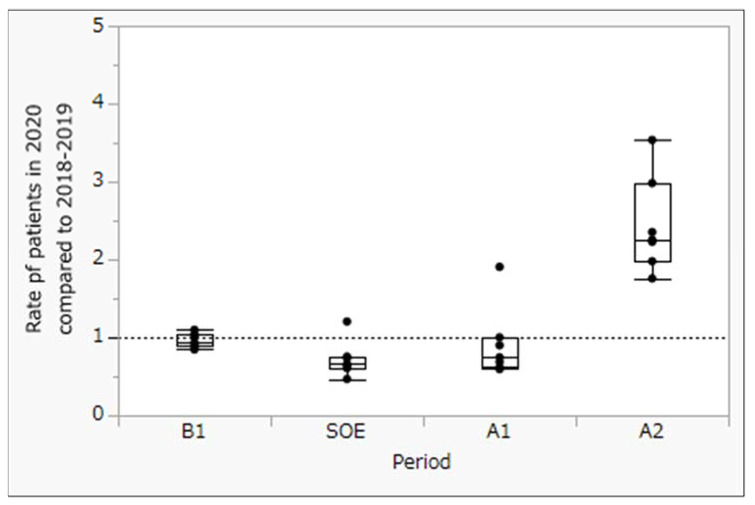
Rate of patients from Tokyo in 2020 compared to 2018–2019 at each time period for calendar week. *p* < 0.05 (Tukey’s honestly significant difference test).

**Figure 4:**
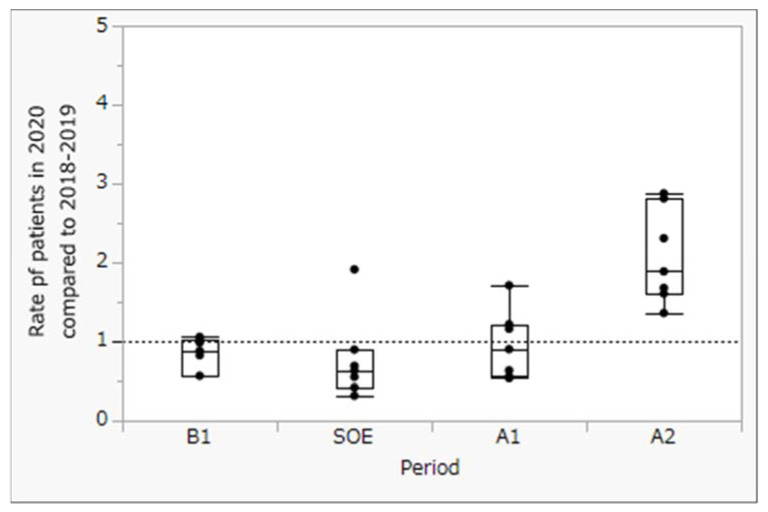
Rate of patients from outside Tokyo in 2020 compared to 2018–2019 at each time period for calendar week. *p* < 0.05 (Tukey’s honestly significant difference test).

To measure the impact of Japan’s state of emergency, we compared the number of weekly patient visits in four seven-week periods: (1) before the state of emergency (B1), (2) during the state of emergency (SOE), (3) immediately after the state of emergency (A1), and (4) at 7 weeks after the state of emergency (A2). Trends in the number of patients with diabetes from inside and outside of Tokyo are shown in Figure 1 and Figure 2, respectively. Moreover, using Tukey’s honestly significant difference test, we compared the rates of patients from inside (Figure 3) and outside of Tokyo (Figure 4) in 2020 to those in 2018–2019 (mean) at each period by calendar week.

This study was approved by the Medical Ethics Committee of Showa University School of Medicine (approval No. 2954). We used JMP 16.2 (SAS Institute Inc. Cary, NC, USA) for all statistical analyses. When the two-tailed *p* value was less than 0.05, we defined the results as statistically significant for all analyses.

## 3. Results

No significant change in the number of patients was seen at 7 weeks before or immediately after the state of emergency. However, a significant increase was seen in the number of patients 7 weeks after the state of emergency was lifted (in May 2020), both from inside and outside of Tokyo.

A comparison of the weekly number of patients for the three periods before and after the state of emergency during 2020 and the previous two years showed no significant differences for patients either inside or outside of Tokyo.

## 4. Discussion

While the results of this study showed a significant increase in visits by patients with diabetes to Showa University Hospital for the first 7 weeks after the COVID-19 state of emergency, compared with the same period in 2018–2019, the number of patients with diabetes did not significantly decrease for 21 weeks, including the 7 weeks before and after the state of emergency. These results suggest that the COVID-19 state of emergency inhibited the provision of continuous diabetes care through delayed doctor visits.

Japan has many public holidays in May, and the effects of these holidays should be noted. A study in southwest Scotland reported that emergency medical visits to a district general hospital showed significantly higher mortality on public holidays, on both weekdays and weekends, although weekend admissions did not generally show higher mortality. However, the mortality rates associated with weekend hospitalizations were not reported [11]. Lapointe-Shaw et al. reported that patients discharged from hospital during the December holiday period were less likely to receive prompt outpatient follow-up and had a higher risk of death or readmission within 30 days [12]. The golden-week holidays usually occur between the end of April and beginning of May in Japan, and the number of hospital visits tends to greatly decrease during this week. According to this schedule, this vacation (about 7 days) may overlap with the effects of the state of emergency. However, as the number of visits by patients with diabetes continued to decrease after the state of emergency was lifted. It is possible that the state of emergency has a long-term effect on consultation behaviours.

Xiao et al. reported that the Chinese lockdown that occurred as a result of the pandemic had a psychological impact on patients with cancer, and it is important to continue psychological support [13]. Infection-control measures during the pandemic are essential to ensure a higher level of preparedness for hospital outbreaks. While major hospitals in Japan were spared from the severe acute respiratory syndrome epidemic, there were substantial differences in emergency infection-control measures, with the differences observed across institutions exceeding those observed across disciplines [14]. Medical institutions showed rural and urban regional differences showing that the health-care system greatly varies according to municipality. Collecting long-term data from a longitudinal study could help to clarify the effects of public holidays (or weekends) and the state of emergency on diabetes patients in Japan. As this was a cross-sectional study, it was not possible to identify the extent of the delays in diabetes treatment. Hanna et al. found that a month-long delay in cancer care/treatment was associated with increased cancer mortality worldwide [15]. Therefore, it is necessary to verify the delay in long-term diabetes treatment and maintain appropriate diabetes treatment during a pandemic.

In this study, our findings suggest that Japan’s state of emergency may not have inhibited hospital visits by patients with diabetes across prefectures. Inoue et al. suggested that personal social capital is associated with continuous medical care among Japanese patients [16]. While the present study focused on urban contexts, the pandemic also affected rural contexts in Japan [17]. Further verification is needed in other regions of Japan.

This study did have some limitations. First, it was based on trends in consultations at a single hospital, so the generalizability of the results could be challenged. This study included information for a large number of patients using data from a single hospital, showing that they refrained from attending medical examinations because of the state of emergency. However, further study, using data from multiple hospitals, is necessary.

## 5. Conclusions

In conclusion, the present findings showed a significant increase in the number of visits by patients with diabetes to Showa University Hospital in the first 7 weeks after the COVID-19 state of emergency, compared with the same period in the previous 2 years. It is also possible that potentially available diabetes care may not have been provided because of the state of emergency; therefore, it is necessary to follow-up patients with diabetes while keeping a close eye on measures to control problems other than infectious diseases.

## Data Availability

The data sets used in our study are not available in a public repository but are available from the corresponding author upon reasonable request.
